# Adult Perspectives on the Long-term Impact of Neonatal Encephalopathy Due to Hypoxia-Ischemia

**DOI:** 10.1055/a-2817-3431

**Published:** 2026-03-13

**Authors:** Corline E.J. Parmentier, Brigitha T.P. van den Broek, Robbert Welles, Evie J.G. Bel, Wouter G. Busstra, Maarten H. Lequin, Niek E. van der Aa, Linda S. de Vries

**Affiliations:** 1 Department of Neonatology, Wilhelmina Children's Hospital, University Medical Center Utrecht, Utrecht, the Netherlands; 2 Department of Neonatology, Willem Alexander Children's Hospital, Leiden University Medical Center, Leiden, the Netherlands; 3 Lived Experience Expert, Utrecht, Netherlands; 4 Edward B. Singleton Department of Radiology, Texas Children's Hospital and Baylor College of Medicine, Houston, Texas, United States

**Keywords:** perinatal asphyxia, hypoxia-ischemia, neonatal encephalopathy, neurodevelopmental outcome, hypoxic-ischemic encephalopathy, patient perspective

## Abstract

Neonatal encephalopathy (NE) following perinatal hypoxia-ischemia (HI) can lead to a wide range of neurodevelopmental consequences, ranging from mild motor problems to severe cerebral palsy, epilepsy, cognitive impairments, and behavioral issues. There is growing recognition of the long-term effects of NE during childhood, but little is known about the outcomes in adulthood, particularly from the perspective of the individuals affected and their families. Existing research primarily relies on outcome measures from neurodevelopmental assessments, which often fail to capture the lived experiences of those affected, limiting our understanding of the meaning and impact of the consequences of NE on their everyday lives. This paper tells the stories of four adults with varying outcomes following NE due to HI, ranging from largely typical development to cerebral palsy to subtle but significant cognitive challenges. Their narratives demonstrate that the long-term consequences of NE are highly variable and influenced by a complex interaction of medical, social, and environmental factors. They shared experiences of challenges, e.g., memory issues, which significantly affect their lives but are not routinely assessed in neurodevelopmental follow-up. Moreover, their narratives underscore that difficulties may emerge or evolve over time, emphasizing the need for ongoing, individualized care. Importantly, the stories of these adults also show that despite the challenges that they have faced and continue to face, they live fulfilling lives that go beyond what results from developmental assessments may indicate. These insights from lived experience experts emphasize the need for a holistic, patient-centered approach in both research and long-term follow-up care.

## Introduction

Neonatal encephalopathy (NE) following hypoxia-ischemia (HI) is an important cause of death and neurodevelopmental impairment. There are numerous studies on the long-term consequences of NE up to school age and adolescence, which demonstrate that beyond motor impairments, these consequences may include cognitive and behavioral problems, epilepsy, or hearing and visual impairments. 1 Nevertheless, the consequences of NE in adulthood are understudied. Importantly, recent studies have demonstrated that the neurodevelopmental trajectories following NE may vary over time, with some difficulties emerging or evolving with age, highlighting that findings at school age are not directly predictive of neurodevelopmental performance in adulthood. 2 3 Moreover, certain consequences of NE that are currently understudied, e.g., memory issues, may significantly hinder independence. 4

In addition to the scarcity of data on the long-term outcomes of NE, these data are predominantly derived from research focused on the patients, without incorporating the perspectives of the individuals and their families. Individuals and their families may prioritize different aspects of functioning than professionals do, and their experiences may reveal challenges that are underrecognized in research or routine care. 5 6 Previous studies in neonatal populations have revealed that the perceptions of patients and parents of neurodevelopmental outcomes differ from those of professionals. 5 7 Therefore, this paper aimed to present the experiences of four adults with a history of NE following HI whose outcomes range from a largely typical development to cognitive challenges, visual impairment, and cerebral palsy, in order to improve our understanding of the lived experiences following NE in adulthood.

## Methods

Two female (B.T.P.B. and E.J.G.B.) and two male (R.W. and W.G.B.) adult coauthors with a broad spectrum of neurodevelopmental outcomes were invited to contribute their lived experiences following perinatal HI. Using open-ended questions, they were asked to describe their birth and NICU course, their developmental experiences, their current adult life, and their expectations for the future.

To provide clinical context without shaping or altering the participants' own accounts, each narrative was introduced with a short summary of neonatal clinical characteristics and neurodevelopmental outcome documented in the outpatient clinic, along with their most recent brain MRI. Clinical and neuroimaging data were retrieved from the Data Registry of Infants with Perinatal Asphyxia, containing clinical and outcome data on subjects admitted to the Neonatal Intensive Care Unit of the Wilhelmina Children's Hospital, the Netherlands, because of perinatal asphyxia from 1990 onward (IRB reference number 22/898). All four adults agreed to the non-anonymous presentation of their medical and imaging data for this paper.

## Githa (32 Years Old, Fig. 1 )

My mother told me that she was 36 weeks pregnant when she had to be admitted to the hospital for CTG monitoring. My older brother was born by cesarean section following placental abruption, and the doctors wanted to prevent this from happening to me. At 42 weeks, labor was induced. Suddenly, my heartbeat dropped and an emergency cesarean was performed. In the operating room it turned out that there was a ruptured uterus. Immediately after my birth the doctors took me away and transferred me to the NICU. My situation was very critical: I had low Apgar scores and the doctors suspected seizures and initiated treatment with several medications. Unexpectedly, an MRI scan, performed a few days after my birth, only demonstrated mild abnormalities. After 13 days of admission, I was finally discharged home.

**Fig. 1:**
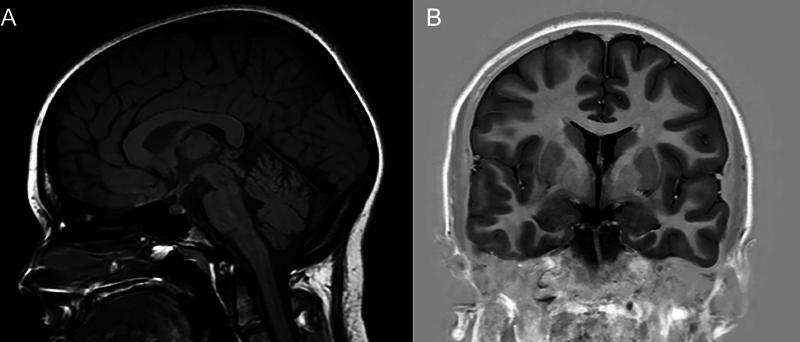
Githa was born by emergency cesarean at 42 weeks gestational age, birth weight 3,490 grams, and a poor start (Apgar 1/1) due to a uterine rupture. Umbilical cord pH was 6.75. Brain MRI at 10 days after birth was classified as normal. At 24 months, her neurodevelopment was normal according to the Griffiths Mental Developmental Scales (GMDS). At 6 years of age, her mother reported concerns about her short-term memory. No abnormalities were noted on brain MRI at 7 years of age. Brain MRI was repeated at 10 years of age, and mild abnormalities in the hippocampi were noted. Brain MRI was performed again at 17 years. In addition to mild abnormalities in the hippocampi, in retrospect, mammillary body atrophy was noted on the sagittal T1-weighted ( **A** ) and coronal inversion recovery T1-weighted images ( **B** ) on the MRI scans performed at 10 and 17 years of age.

Initially, I did not experience any specific consequences of my difficult birth. I was treated like a normal child, and went to a mainstream school. When I was around 10 years old, I started to notice having more difficulties at school compared to my peers. I had to study hard, and it took me a lot of time to do my homework. However, as I grew up, I also increasingly experienced issues with my memory. My mother reached out to the neonatologist to inform whether this could be a consequence of my birth history. Years later, it turned out that I have atrophy in brain areas related to memory ( Fig. 1 ).

Nowadays, as a 32-year-old adult, I still struggle with my memory. Although I am able to live on my own, I have to use a lot of tricks to help me from forgetting everyday tasks, like getting groceries or putting on the dishwasher. For example, I set alarms and have a checklist by my door with the things that I should take with me when leaving home.

My parents have always been very supportive, and treated me like a normal child. However, they told me that they had a difficult time during the first year after my birth. Initially, my father had no memory of all the events that occurred immediately after my birth, and my mother told me that it took her a year to emotionally heal from her experiences in the NICU.

Looking back at my youth, I think I would have benefited from more support at school, with more time allowed for studying and exams. I studied very hard, but I clammed up during exams and struggled with my memory. My school tutors did not really know how they could help me. However, I am very thankful for all the support that I have received, particularly from my parents and my brother. Recently, I got into a relationship. My boyfriend accepts me for who I am and helps me with my memory problems.

I believe that my birth history has also brought me something. Nowadays, I work with elderly people who have Alzheimer's disease, and I believe that due to my own difficulties with my memory I have become particularly good at taking care of them. I also work very hard and don't give up easily. After years of saving money, I was able to live by myself and decorate my own house. Recently, I made a 3-week trip to Japan with my brother. Looking back at these accomplishments makes me extremely proud.

## Robbert (29 Years Old, Fig. 2 )

I was born at home, with an unexpected poor start after an uncomplicated pregnancy, and had to be transferred to the NICU because of seizures and need for ventilatory support. I was quite a large baby between all the preterm infants according to my parents. The first week of my admission I received a lot of anti-seizure medication. After 1 week, the medication could be weaned off, and my parents saw me opening my eyes. After 3 weeks of admission, I was discharged home.

My birth complications ultimately resulted in cerebral palsy, and I have received a lot of therapy while growing up: physical, occupational, and speech therapy. According to my parents, I was a passive baby during the first few months. Music and singing helped; it immediately grabbed my attention. After those initial months, I developed into a happy child. Although my motor, cognitive, and speech development was slower than my peers and my parents had to practice a lot with me, I did learn how to roll over, sit, crawl, and stand.

At first, I did not really experience major difficulties while growing up. However, the right side of my body did not function as well as the left side, I stumbled easily, and I struggled with my writing and pronunciation. My parents also noticed me having problems with my short-term memory and the processing of information. They often had to repeat things for me to get the point. However, what really made an impact on me was the diagnosis of epilepsy when I was 10 years old.

Nowadays, as a 29-year-old adult, I live with five others in a supported living accommodation, where I have my own bedroom, bathroom, and kitchen. I do my own laundry, buy my own groceries, and we take turns in cooking for each other. I work 5 days a week at the library, under the guidance of staff from my healthcare facility. Mostly I get there by bus, but sometimes I walk. Outside of work I enjoy going out for dinner, spending time on the computer, visiting theme parks, or going to a club or festival with my brothers. I am currently working hard to become more independent. For instance, I am practicing using public transport so I can go to the hairdresser on my own.

Although I was diagnosed with epilepsy early in life, I was fortunate to outgrow it. However, as an adult, I still deal with long-term consequences of my birth complications, especially memory impairments. Remembering everyday things requires a great deal of effort. I often forget whether I have already eaten, to change my clothes, or what plans I have made for the coming days. It can be mentally exhausting, and I have learned that I need to slow down regularly to give my brain some rest. Time management is another challenge: I frequently lose track of time and end up staying up too late. What really helps me cope are consistent routines and the use of a digital assistant to keep things organized.

Despite these difficulties, I have grown in ways I am proud of. I have become highly skilled with computers and developed strong social abilities. As a child, I had to work hard to improve my communication skills, but now I genuinely enjoy connecting with others. I love getting to know people, engaging in political discussions, and even giving speeches or presentations.

I am deeply thankful for the people who surround me: my parents, brothers, roommates, friends, and the residential support workers. My parents have put a lot of effort in helping me grow and learn. They recognized early on that my cognitive abilities were held back by my motor impairments, and helped me overcome these barriers, for instance by teaching me how to use a computer mouse. My brother has always been both my biggest support and my favorite playmate. We have always had a special connection, and that bond remains strong today. Together we created a documentary about my life, which was even featured on national television. I am very proud of this accomplishment, and of my ability to live as independently as possible.

## Evie (22 Years Old, Fig. 3 )

My mother was referred to the local hospital 22 years ago during a routine prenatal check-up because of concerns about my slow heart rate. As soon as she lay down on the hospital bed, her waters broke, and an emergency cesarean section was performed. After my birth, I was immediately transferred to the neonatology department and thereafter to the level-three NICU, without my parents being able to see or hold me. In the NICU, the doctors explained to my parents that I had suffered perinatal asphyxia and the MRI showed brain injury. A lot of uncertainties and discussions about the long-term consequences of my birth complications followed. After 3 weeks, with my parents by my side throughout the whole hospital stay, I was finally discharged home.

A lot of examinations followed during the first year after my birth. An MRI scan, performed when I was 3 months old, demonstrated brain atrophy in the areas that were related to my visual functions, and I received a lot of therapy to support my development. Growing up, I remember often losing my friends because of my visual impairment, and being a lot slower at things than the other children. School took a lot of energy out of me. Fortunately, things got a lot better when I switched to a special school for children with visual impairments when I was 6 years old.

I still experience the consequences of perinatal asphyxia. I am not able to see the lower part of my visual field, which results in me often stumbling over things and not being able to read subtitles when watching the news or a movie. I also find it difficult to estimate the movements of an object, and I can't find my way in new surroundings. I remember getting lost on a holiday, only after a 5-minute walk from the apartment where we stayed. I also struggle with eye–hand coordination. For example, it took me years before I was able to tie my shoe laces, despite knowing the practical steps in my head. I also face difficulties with my short-term memory. Therefore, I write down a lot of things and try to do everything with my full attention.

Despite these difficulties, I am very thankful for my life and the support that I have received and that I am receiving right now. I am very thankful for my friends: for their help, their efforts to look beyond my impairments to get to know me, and for our valuable memories. I am thankful for the support from the health care workers and my teachers: I obtained a degree as a Legal Insurance and Human Resources Services Specialist, and within a few months I will go to Law School. And I am thankful for my parents and their endless investments in me, including their private lessons and taking me to physical therapy. From the notes in my mother's diary, I have learned that they had a very difficult time during the first year after my birth. I wish there would have been a way for me to support them during this difficult period. I am particularly thankful that my parents helped me learn how to write. Writing is everything to me: I write songs, stories, and poetry, and I have written two books which also discuss my visual impairment. These books emphasize that we all have our impairments and strengths, and that we become perfect by helping each other and combining our talents. It is my biggest dream to become a professional writer, and to write a book which will be made into a movie and become available in all languages.

## Wouter (27 Years Old, Fig. 4 )

I was born at home with a poor start because of shoulder dystocia, with Apgar scores of 0, 1, and 2. While the midwife stayed with my mother, my father had to wait with me for the ambulance while I showed no signs of life. When the ambulance arrived at the NICU, my father and our priest prayed for me. According to my father, that was the moment that I started breathing. The doctors spoke of a miracle.

During my NICU admission, my parents were prepared for a bad outcome. I had to receive mechanical ventilation and developed seizures. However, I recovered and after 10 days I could be discharged from the NICU.

Fortunately, I did not develop any major impairments. At 2 years of age, I was even ahead of my peers in terms of development, and I was discharged from follow-up. However, memory problems have always been an important theme throughout my life. At primary school, I managed to hide these struggles. I relied on logic instead of learning. However, this became more difficult at high school, particularly with learning new languages. In this period, I was diagnosed with ADD. Methylphenidate improved my learning, although I had to switch to an extended-release tablet, as I always forgot to take the second dose. When I started my study at the university of applied sciences, I stopped my medication, because I didn't want to rely on it in order to function.

Nowadays, I am very happy with my life. I am happily married, living in a lovely home, and working in project management, where I contribute to the energy transition. I also enjoy staying active through sports. Despite this, I still face challenges with my memory. Both at work and at home, I need to take time to write down appointments and tasks to stay organized. My wife supports me by helping me remember things, which I really appreciate. To manage these difficulties, I rely heavily on reasoning and logic, which reduces the need to memorize everything. Over time, this has actually strengthened my logical thinking skills. While I struggle with short-term memory, my long-term memory is excellent: I still recall things I learned about geography back in primary school.

There has been a lot of frustration from those around me regarding my memory issues. Even though I now feel that my friends and family have come to accept it, I have often struggled with the feeling that I should have tried harder to improve my memory. Until this year, my parents and I did not know that these issues were related to my birth history. Knowing the cause has brought clarity. Not just for me, but also for those close to me.

I am deeply grateful for the life I have. Every day feels like a gift. Considering the early expectations that I might face severe disabilities, I am thankful to have a well-functioning body and to live such a fulfilling life. I am especially proud of my wife and the happy life we have built together. If I could give one piece of advice to other adults with complications around the time of birth, it would be to focus on your strengths and opportunities rather than your limitations: “Comparison is the thief of joy” (Theodore Roosevelt). At the same time, it is important to be aware of the potential long-term consequences of conditions like perinatal asphyxia. We cannot change the past, but we do have a choice in how we respond to its consequences.

## Discussion

Four adults with NE following HI tell their stories and show that they have faced and are still facing different issues related to their birth complications. Despite the differences in their neurodevelopmental trajectories, their narratives also reveal important similarities. The non-motor consequences of NE often only became apparent later in development. Moreover, all four report on short-term memory problems, with a significant impact on their lives during childhood and adulthood. Yet, their stories also powerfully embody gratitude and resilience.

Although there is growing interest in the outcomes of perinatal brain injury into adolescence and adulthood, large-scale studies on the long-term impact of NE following HI remain scarce. A study from Sweden reported that infants born with a poor condition (as indexed by a 5-minute Apgar score <7) without NE had worse measures of education, income, and socioeconomic position in adulthood than those born in good condition. 8 Those with NE were more likely to be in the lowest income quartile and to have no income from work. However, 32% of this group attended university and nearly two-thirds gained employment in early adulthood.

The few studies that have evaluated the consequences of NE in adolescence demonstrated that, beyond motor problems, these can include a variety of cognitive issues, including problems with executive functions, attention, and auditory working memory. 6 9 Particularly infants with a pattern of watershed injury on brain MRI were considered at risk for cognitive impairments later in life. 10 11 In accordance with the narratives in this paper, these impairments were often not yet apparent in early childhood. The concept of “growing into deficits” following perinatal brain injury is increasingly recognized and underscores the need for long-term neurodevelopmental surveillance. 2 3

**Fig. 2:**
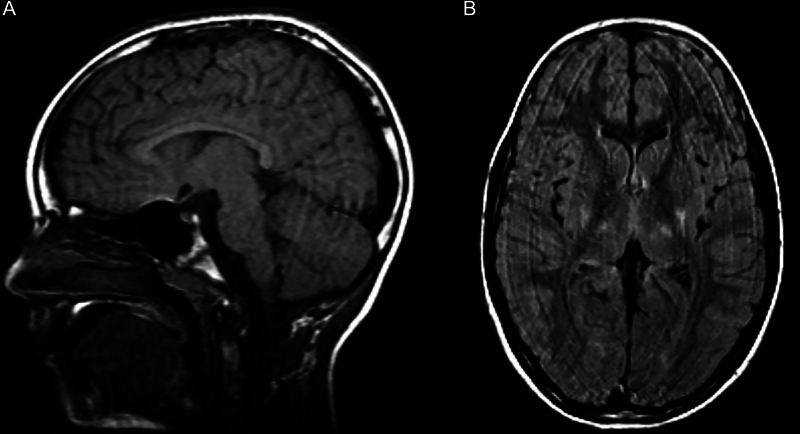
Robbert was born following a planned home delivery at 41 + 3 weeks gestational age, birth weight 3,350 grams and Apgar scores 4/5 at 1 and 5 minutes. He was admitted with seizures, which were treated with phenobarbital, clonazepam, phenytoin, and lidocaine. A brain MRI was performed on day 7, showing signal abnormalities in both ventrolateral thalami and lentiform nuclei. He developed a mild athetoid cerebral palsy. At the age of 2 years, Griffiths Mental Developmental Scales (GMDS) scores were locomotor 15.5 months, personal–social 22 months, hearing–speech 20 months, eye–hand 19 months, performance 20 months. At 10 years of age he was diagnosed with epilepsy, for which he initially received valproic acid, and later switched to carbamazepine. MRI at 10 years showed mammillary body atrophy (sagittal T1 weighted image) ( **A** ) and gliosis in the basal ganglia and thalami (axial FLAIR image, **B** ).

**Fig. 3:**
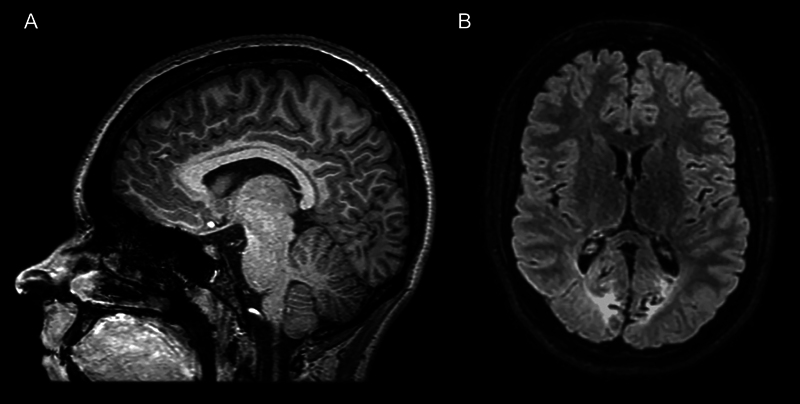
Evie was born at 40 weeks gestational age by emergency cesarian section because of fetal bradycardia with Apgar scores of 7/7 at 1 and 5 minutes, umbilical cord pH 7.20, and birth weight 3,370 grams. There was thick meconium-stained amniotic fluid. She was intubated because of respiratory insufficiency and hypotonia at 20 minutes after birth and was subsequently admitted to the neonatal intensive care unit. There she developed seizures within the first hour of admission, which were attributed to subacute perinatal asphyxia and treated with phenobarbital, lidocaine, and midazolam. Brain MRI performed at 9 days after birth demonstrated signal abnormalities in the occipital lobes, and she developed a quadrantanopia. Griffiths Mental Developmental Scales (GMDS) scores at 3 years of age were locomotor 38 months, personal–social 32 months, hearing–speech 32 months, eye–hand 28 months, and performance 36 months. At 16 years of age, a brain MRI was performed again, demonstrating mammillary body atrophy on sagittal T1-weighted imaging ( **A** ) and bilateral atrophy and gliosis in the occipital lobes on axial FLAIR imaging ( **B** ).

**Fig. 4:**
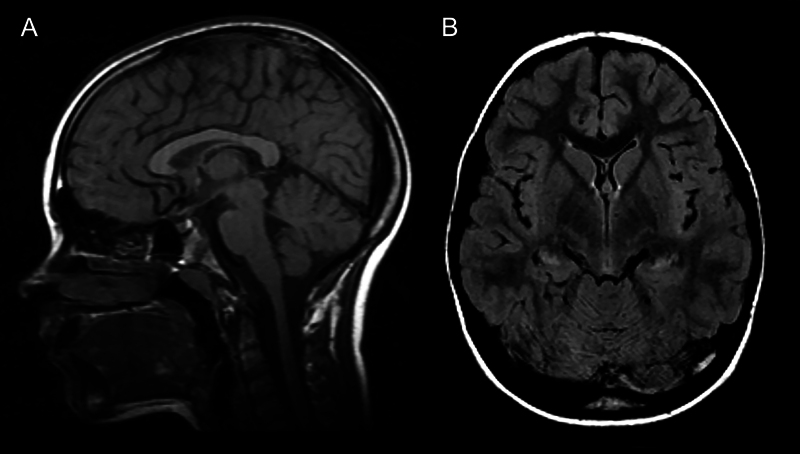
Wouter was born after a planned home delivery at 40 weeks gestational age with a birth weight of 4,900 gram, and a poor start (Apgar 0/1/2 at 1, 5, and 10 minutes) due to a shoulder dystocia. He was admitted to the NICU and developed seizures, which were treated with phenobarbital, phenytoin, and midazolam. Besides a small subdural hemorrhage, no other abnormalities were noted on an MRI performed on day 10. At 24 months, the Griffiths Mental Developmental Scales (GMDS) score demonstrated normal neurodevelopment (locomotor 26 months, personal–social 24 months, hearing–speech 24 months, eye–hand 24.5 months, and performance scale 23.5 months). Brain MRI performed at 10 years of age demonstrated mammillary body atrophy on sagittal T1-weighted imaging ( **A** ) and mild gliosis in the hippocampi on axial FLAIR imaging ( **B** ).

All four adults in this paper describe problems with their memory. Although memory issues have long been related to hippocampal atrophy following HI injury, there is now also emerging attention to mammillary body injury in perinatal asphyxia. 1 4 12 These memory impairments can be challenging to detect, particularly in early childhood. They can be mistaken for benign forgetfulness or inattentiveness, and children may develop compensatory strategies to mask their difficulties, as illustrated by the story of Wouter. However, such memory deficits can have profound and lasting consequences, potentially hindering independence in adulthood. This underscores the importance of thorough evaluation of memory abilities in children with a history of NE and development of reliable tests to assess memory function in early childhood. 13 14 Moreover, the story of Wouter highlights the significant value that an explanation of these difficulties can provide to individuals and their families, even when no curative intervention is available. Therefore, it is important to carefully assess the limbic system, including the mammillary bodies and the hippocampi, on brain MRI, using scanning protocols with sufficiently thin slices to enable detection of injury (see Figs. 1 – 4 ). 12

It is important to realize that existing research on the long-term consequences of perinatal brain injury predominantly focuses on biomedical aspects, using standardized neurodevelopmental tests to quantify impairments and functional capacities. Although these tools provide important clinical information, they capture only a narrow aspect of the lived experience. Furthermore, outcome classifications have been shown to correlate poorly with parental perspectives, and clinical trials use composite outcomes that equate death and various comorbidities. 5 There are widely acknowledged concerns about the use of these composite outcomes, including their limited value for parents, a lack of clinical utility, and the risk of either inflating or diluting effect sizes. 15 The narratives in this paper illustrate that adult outcomes are not solely defined by neurological test scores or diagnostic labels, but rather emerge from a dynamic and complex interplay of medical, social, and environmental factors, within each individual's life context. A sole focus on biomedical endpoints is likely to result in missed opportunities to improve care by overlooking both the challenges that necessitate ongoing support and the strengths that enable individuals to thrive. Improving the relevance and impact of research and follow-up care requires a more holistic approach, and engagement with experienced experts to determine outcomes that are important to them.

The International Classification of Functioning, Disability and Health (ICF) offers a valuable framework for situating health within a broader social–ecological context. 16 It could guide a shift toward a more comprehensive approach to study outcomes, for instance, by using the F-words: function, family, fitness, fun, friends, and future. 17 Patient-reported outcome measures allow individuals and families to report on aspects of health, functioning, and well-being that may not be visible to clinicians and can capture domains such as fatigue and social inclusion. 18 Methodologically, incorporating patient-centered approaches such as Desirability of Outcome Ranking can provide more nuanced ways to analyze and present outcomes in trials, by ranking or weighting outcomes based on their desirability or relevance to individuals and families, rather than treating all events within a composite outcome as equally important. 19

The adults highlight the importance of social support in enabling them to succeed in their careers and personal pursuits. Despite the challenges, all four individuals demonstrated that early difficulties could evolve into strengths, whether through enhanced logical reasoning, empathetic capacities, or creative approaches to share their stories and encourage others. This emphasizes that supporting strategies for individuals with a history of NE should go beyond medical treatments to include tailored coping mechanisms that enhance support and leverage individual strengths. These may range from early motor and neuropsychological interventions to the use of assistive technology. 20 21 22 Moreover, there is a critical need for broader societal awareness of the long-term impact of NE, particularly among educators, public authorities, and the general public, to foster more supportive environments for affected individuals.

Importantly, this paper presents narratives from a select group of four individuals who were able and willing to share their stories. A recent study on children ≥8 years of age treated with therapeutic hypothermia for NE has demonstrated that these children and their families encounter a broader range of everyday challenges beyond those described in this paper, including impairments in attention, processing speed, and impulse control. 6 Large-scale studies are needed to understand the breadth of consequences associated with NE in adulthood. Furthermore, the improvements in neonatal care over recent decades, including the introduction of therapeutic hypothermia and changes in seizure management, with discontinuation of medication at discharge, should be taken into account when relating these accounts to contemporary cohorts.

In conclusion, the narratives in this paper illustrate that the consequences of NE in adulthood are variable and can evolve across development, with memory difficulties emerging as particularly impactful yet often underrecognized challenges. These accounts underscore the importance of long-term neurodevelopmental follow-up and the use of outcome measures that reflect lived experiences rather than solely biomedical endpoints. The stories also reveal the significant value of social support, adaptive strategies, and personal resilience in shaping adult functioning. Although this paper only represents the story of four individuals, their narratives emphasize the importance of listening closely to lived experiences, and integrating these perspectives into large-scale studies to fully comprehend the impact of NE in adulthood.
